# Involuntary placement of a mentally ill person in a psychiatric hospital and care institution

**DOI:** 10.1192/j.eurpsy.2021.1022

**Published:** 2021-08-13

**Authors:** A. Arold, J. Kostomarova

**Affiliations:** 1 C, Marienthali Kliinik, Tallinn, Estonia; 2 Law, University of Tartu, Tartu, Estonia

**Keywords:** involuntary placement, mental health, psychiatry, mental health legislation

## Abstract

**Introduction:**

In the mental health area, the most problematic issues are the involuntary placement of the mentally ill in closed institutions, both under civil and criminal proceedings, and their involuntary treatment. Despite the international efforts of harmonizing measures, the nature and practice of the services still vary from country to country.

**Objectives:**

To analyse involuntary placement of persons with mental disorders in closed institutions under civil and criminal proceedings, which include both psychiatric hospitals and care institutions.

**Methods:**

Review and analysis of regulations and practice of involuntary placement of a person with a mental disorder in a closed institution in the context of Estonian, Finnish, Russian, and English law, health care and social system.

**Results:**

Estonian, Finnish, Russian, and English law distinguish between criminal and civil proceedings regarding involuntary placement of a mentally ill person in a closed institution. However, specifics of the proceedings are different among the countries, e.g. judicial involvement, and deadlines. Also, the provision of forensic mental health services differ among these countries, e.g. in Estonia offenders and non-offenders are kept separately, whilst in England and Russia patients are not distinguished so strictly.

**Conclusions:**

The distinction between involuntary placement of the mentally ill in criminal and civil proceedings is distinguished primarily for the reason that in one case the risk arising from the person is directly realized by committing an unlawful act and in the other case the risk arising from the person is directed at themselves or is not qualified as an unlawful act.

